# Unscreened Water-Diversion Pipes Pose an Entrainment Risk to the Threatened Green Sturgeon, *Acipenser medirostris*

**DOI:** 10.1371/journal.pone.0086321

**Published:** 2014-01-15

**Authors:** Timothy D. Mussen, Dennis Cocherell, Jamilynn B. Poletto, Jon S. Reardon, Zachary Hockett, Ali Ercan, Hossein Bandeh, M. Levent Kavvas, Joseph J. Cech, Nann A. Fangue

**Affiliations:** 1 Wildlife, Fish, and Conservation Biology Department, University of California Davis, Davis, California, United States of America; 2 Department of Civil and Environmental Engineering, University of California Davis, Davis, California, United States of America; The Australian National University, Australia

## Abstract

Over 3,300 unscreened agricultural water diversion pipes line the levees and riverbanks of the Sacramento River (California) watershed, where the threatened Southern Distinct Population Segment of green sturgeon, *Acipenser medirostris*, spawn. The number of sturgeon drawn into (entrained) and killed by these pipes is greatly unknown. We examined avoidance behaviors and entrainment susceptibility of juvenile green sturgeon (35±0.6 cm mean fork length) to entrainment in a large (>500-kl) outdoor flume with a 0.46-m-diameter water-diversion pipe. Fish entrainment was generally high (range: 26–61%), likely due to a lack of avoidance behavior prior to entering inescapable inflow conditions. We estimated that up to 52% of green sturgeon could be entrained after passing within 1.5 m of an active water-diversion pipe three times. These data suggest that green sturgeon are vulnerable to unscreened water-diversion pipes, and that additional research is needed to determine the potential impacts of entrainment mortality on declining sturgeon populations. Data under various hydraulic conditions also suggest that entrainment-related mortality could be decreased by extracting water at lower diversion rates over longer periods of time, balancing agricultural needs with green sturgeon conservation.

## Introduction

Sturgeons, family Acipenseridae, are among the oldest bony fishes in existence and have been successful for millions of years with a life-history strategy characterized by longevity, delayed maturation, long breeding intervals and iteroparity. Recently, however, sturgeon populations have been particularly vulnerable to over-harvesting, habitat alteration, and habitat loss [1], [2]. Declining sturgeon populations worldwide have reached protected status [3], with 15 of the remaining 25 species listed as critically endangered on the International Union for Conservation of Nature Red List [4]. Among these, green sturgeon (*Acipenser medirostris*) occur in coastal waters from Alaska to Mexico, and the Southern Distinct Population Segment (DPS), which spawns only in the Sacramento River basin, was listed as threatened under the US Endangered Species Act in 2006 by the National Marine Fisheries Service, due to reproductive isolation, limited spawning habitat and low estimated population abundance [5]. It is therefore vital to evaluate unquantified green sturgeon mortality risks, such as those posed by the thousands of agricultural water diversions on the Sacramento River, and develop management strategies to help ensure the persistence of this imperiled fish.

**Figure 1:**
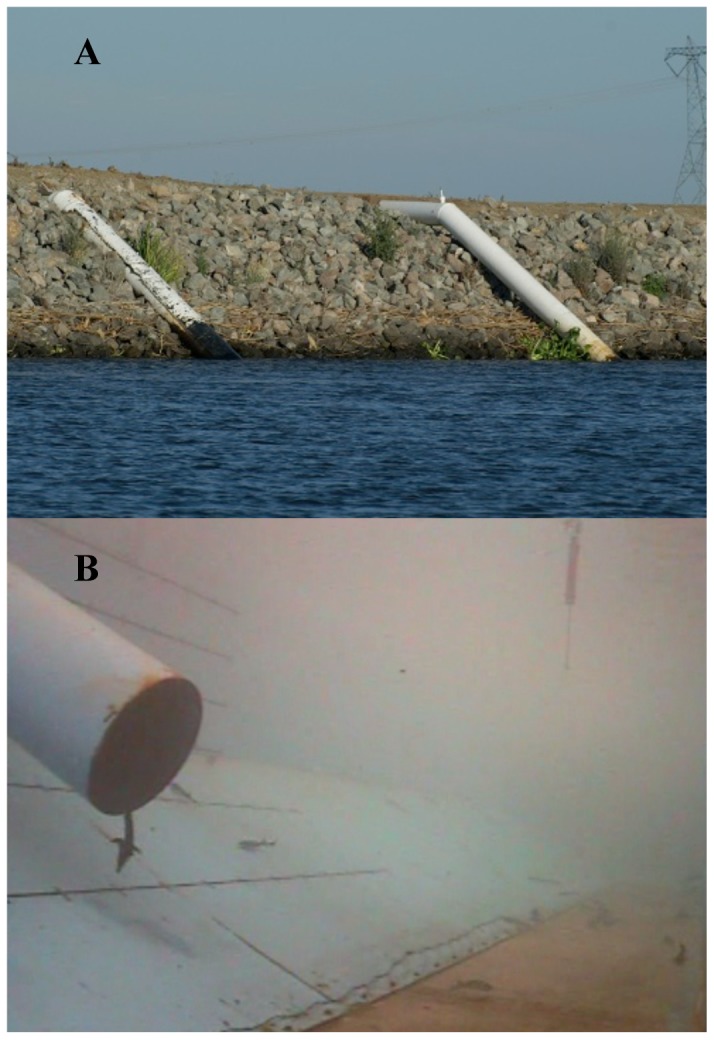
Images of (A) water diversion pipes located in the lower Sacramento River, California, USA and (B) green sturgeon swimming in the flume with a green sturgeon drawn (entrained) into the water diversion pipe during an experiment, recorded with an underwater camera positioned 2.1 m downstream of the pipe.

Unscreened agricultural water-diversion pipes lining levees and riverbanks represent a significant threat to fish, unless individuals exhibit avoidance behavior. Fish can be drawn into these pipes (a process termed ‘entrainment’), and either killed directly by physical damage from the pumps, or indirectly through stranding in the seasonally irrigated canals, ditches, and fields where the water diversions empty. All entrained fish, regardless of the mechanism, are ultimately lost from the population. The threat of fish entrainment to other migratory and resident species is well-recognized worldwide [6]. In California the actual number of out-migrating juvenile green sturgeon entrained into water diversions is unknown [7], but with over 3,300 water diversions operating in the Sacramento-San Joaquin Watershed (Figure 1A) and with over 98% of these unscreened [8], there is potential to entrain juvenile green sturgeon [5](Figure 1A). There have been few observations of green sturgeon entrainment on the lower Sacramento River [9], but the fish's susceptibility to entrainment has never been directly quantified. Undocumented seasonal water diversions also occur in some parts of California that may be adding to fish entrainment risks [10], but because they are ‘hidden,’ estimating their potential effects is challenging. Green sturgeon entrainment risk is further increased because they spawn in the upper reaches of the Sacramento River (late-April to June) [11], and the downstream migration of juveniles (May to August) [12], coincides with peak agricultural water extraction (April to September) [13]. Juvenile green sturgeon may reside in freshwater for 0.5 to 1.5 years [14], [15], making repeated interactions with active unscreened water diversions possible. Therefore, the goal of this study was to identify green sturgeon's entrainment susceptibility to unscreened water diversions operating over a range of hydraulic conditions.

## Materials and Methods

### Ethics Statement

All animals were handled according to the UC Davis Institutional Animal Care and Use Protocols (IACUC # 15836). At the completion of each swimming experiment, entrained fish as well as those remaining in the flume were quickly (ca. 5 s) transferred to recovery tanks to be counted, weighed and measured, and sacrificed in buffered MS-222 anesthetic bath.

Juvenile green sturgeon entrainment susceptibility was evaluated in a large (>500-kl) river-simulation flume [16] over a range of realistic Sacramento River flow velocities (sweeping velocities) and water-diversion rates [9] through an unscreened, 0.46-m-diameter diversion pipe. Experiments were performed using combinations of 0.15, 0.38, and 0.61 m/s sweeping velocities with 0.42 and 0.57 m^3^/s water diversion rates, as well as a 0.15 m/s sweeping velocity with a 0.28 m^3^/s water diversion rate. Experiments were designed to simulate a common “over-the-levee” style agricultural water diversion [9] (Figure 1A) including a simulated riverbank with an angled bank (ramp) that was located down the length of the flume at a 26.6° decline from one sidewall to the base of the flume. The unscreened diversion pipe was mounted near the center of the flume parallel to the angled ramp with its base located 0.3 m above the ramp to simulate a typical irrigation pipe (Figure 1B). Flume sweeping velocities were controlled using variable-speed pumps. Water passing through the flume flowed into an in-ground tail tank by head difference, either traveling through the diversion pipe or by passing through a downstream weir. Fish were restricted to swimming in the main channel by upstream and downstream stainless-steel screens (0.6-cm mesh), and fish entrained through the pipe were captured in an extractable underwater fyke trap with a mesh bag. This design minimized injuries to entrained fish by preventing fish passage through water pumps. Flume water temperature (19.4°C±0.33 SE), dissolved oxygen concentration (11.58 mg/l±0.18 SE), illuminance (1190 lux±0.38 SE, measured 1.2 m above the water surface), ammonia concentration (undetectable, 0.00 mg/l), and pH (8.01±0.01 SE) were measured at the start and end of each experiment. Flume water was drained and refilled weekly with non-chlorinated, well water.

Due to the threatened status of wild southern DPS fish, green sturgeon (Northern DPS), 26–36 weeks of age, were spawned from 3 actively spermiating males and a single female using established tank spawning methodologies [17], [18], [19]. During the experimental period, fish were held in one of three 7340-l flow-through circular tanks equipped with non-chlorinated, air-equilibrated well water (18°C, pH: 8.0, dissolved oxygen: 7.5–9.5 mg/l, 0 ppt salinity) and fed a dry pellet diet (SilverCup™) daily to satiation. Mean green sturgeon fork length was 34.9 cm (SE, 0.6) and mass was 207.7 g (SE, 12.6, Ohaus balance model: SC4020). At the start of each experiment, 60 naive fish were transferred to the flume in aerated coolers and placed into a submerged release cage, located 9.3 m upstream from the diversion pipe, via a 2.1 m long, 15.2-cm diameter PVC tube for a 30-min acclimation period. After the acclimation period the pumps were initiated and the hydraulic conditions of each treatment were rapidly stabilized (ca. <1 min). The downstream wall of the cage was then opened remotely, which demarcated the start of each experiment, and hoisted out of the water for the duration of the experiment. Fish swimming behaviors and entrainment events were recorded continuously during an hour-long experimental period, using five video cameras. Water diversion rates were measured using a digital transit time flow meter (Polysonics, DCT7088). Each flow combination was replicated 6 times (42 experiments, total) and tested in randomized order during the experimental period to control for increases in fish age and size. At the completion of each swimming experiment, the underwater extractable cage and mesh bag that contained the entrained fish was hoisted out of the water and fish were quickly (ca. 5 s) transferred to a recovery tank. Fish remaining in the flume were collected using a 3.7 m by 3.0 m seine net and placed into a separate recovery tank. Fish were then sacrificed in buffered MS-222 anesthetic bath, counted and measured (fork length in cm and mass in g).

Fish were filmed in the flume using video cameras (Sony model: CCD-TRV108, Canon model: ES200A, and Speco model: CVC 627) and five DVD-R's (Panasonic model: DMR-EA18K) to continuously record each experiment. Cameras were mounted either underwater on the flume's sidewall using 45.4-kg-force magnets, on tripods outside of the flume, or above the flume's swimming channel. All cameras were directed at the diversion pipe's inlet to record entrainment events. One of the underwater cameras inside the flume was located 2.4 m downstream and at the same depth as the pipe inlet and the other was positioned directly across from the diversion pipe, 1.5 m above the bottom of the flume. Two cameras viewed the diversion pipe inlet through acrylic windows located 0.61 m upstream and 0.61 m downstream from the pipe inlet in the sidewall of the flume. One camera was mounted 4 m above the surface of the water, providing observation from directly above the diversion pipe inlet. A clear-plastic view plate (1.22 m×1.22 m) was floated below this camera to reduce water-surface-related distortion providing a clear view of the water diversion pipe.

The timing, starting locations and resultant water velocities were determined for each fish entrainment event using video recordings and J-watcher software (v 1.0) [20]. The timing and number of successful pipe passage events (defined as each time a fish traveled past the water diversion pipe from upstream to downstream, or from downstream back upstream, at any distance without becoming entrained) were analyzed. The mean number of fish that successfully passed the diversion pipe or that became entrained into the pipe were calculated in 10-min intervals, and were also used to calculate mean entrainment susceptibility risk for each experiment. Because individual fish could not be identified, differences in pipe passage rates between individual fish were unknown. The mean entrainment risk per pipe passage was calculated for each flow combination by dividing the number of entrained fish by the observed number of pipe passages that occurred during the experiment (including both successful passage+passages resulting in entrainment), multiplied by 100. Fish were considered to have ‘encountered’ a water diversion when swimming within 1.5 m of the inlet (the maximum distance from it when passing it in our experiments). The percentage of fish lost to entrainment following repeated encounters with unscreened diversion pipes was estimated by repeatedly multiplying the product of a variable (starting at 100) by the calculated fraction of fish diverted during pipe passage and summing the resulting differences between the starting value and product for each iteration (representing repeated pipe passages). As an example, after 3 pipe passages, with 22.3% entrainment risk per passage, the percentage of fish entrained was estimated to be 53%, also calculated as (100*(1-(1-0.223)^3^).

The starting locations of the first 10 fish entrainment events from each experiment were used to calculate mean fish entrainment distance. Still images of fish entrainment events were created (Sony, Movie Studio HD platinum 1). Images of the fish's position relative to the diversion pipe were captured from the video at the moment when the fish started to become entrained into the diversion pipe, indicated by a change in the movement direction or velocity of the fish as it approached the diversion pipe (i.e., entrainment-starting location). Still images of the entrainment-starting locations were made for each fish-entrainment event from the overhead and side window cameras, allowing distances to be measured from the top and front perspective. The distance and angle from the center of the pipe's inlet to the center of the fish's head were measured in each image using ImageJ software [21]. The combined measurements allowed the fish entrainment-starting locations to be defined in three-dimensional space relative to the center of the diversion pipe's inlet. Because the camera's perspective distorted the true measurement distances, measured fish entrainment-starting distances were modified by empirical camera correction formulas. To create the correction formulas, post-experiment ratios of observed to actual distances were calculated from still images of a suspended PVC pipe grid, at 15.2-cm intervals from the center of the pipe inlet. Once fish entrainment-starting locations were identified through video analysis, the flow combinations (sweeping flow, diversion rate, and water depth) were recreated in the flume to measure the exact 3-dimensional velocities (at 25 Hz, 3-D SonTek ADV probe, ±1%) where each entrainment began.

Data were analyzed using ANOVA models and Tukey's post-hoc tests with SAS 9.2 software. Significance was set at alpha ≤0.05. Differences in fish entrainment counts between flow combinations were analyzed using a two-way ANOVA with a Poisson distribution. Differences in the mean successful pipe passage counts, percentage of fish that became entrained per pipe passage event, fish entrainment-starting distances, resultant water velocities at entrainment starting locations, and fish entrainment event durations were analyzed between flow conditions using two-way ANOVAs with normal distributions. Percentages were arcsine-transformed prior to ANOVA analysis to normalize the data. Fish body size can influence maximum swimming speed [22], and therefore mean fork lengths and masses of entrained and non-entrained fish were compared at each flow combination using t-tests to determine if fish size affected entrainment risk. Fish fork lengths and masses were compared with an ANOVA to identify potential differences in fish size among flow combinations.

## Results

**Figure 2:**
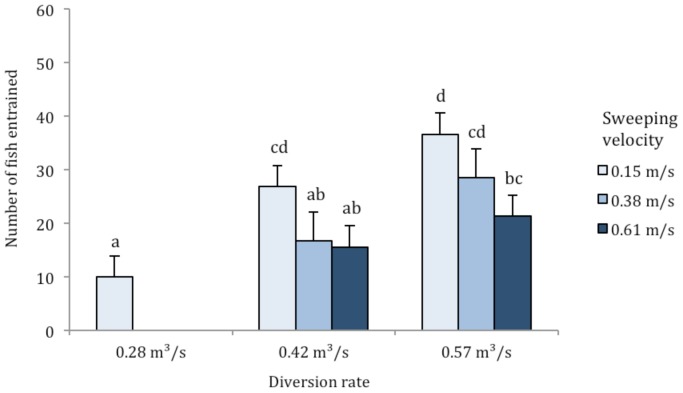
Mean ± SE number of green sturgeon entrained through the unscreened diversion pipe at sweeping velocities of 0.15, 0.38 and 0.61 m/s and water diversion rates of 0.28, 0.42 and 0.57 m^3^/s. Significant differences in the number of fish entrained at different flow combinations are marked with different letters (*P*≤0.011 for all significant pairwise comparisons).

Overall, a surprisingly large percentage of sturgeon became entrained through the unscreened pipe, ranging from 26% entrainment to 61% entrainment at the most challenging flow combination (Figure 2). Fish entrainment was significantly higher at lower sweeping velocities (*F*_2,35_ = 22.4, *P*<0.001) and at higher water diversion rates (*F*_2,35_ = 49.9, *P*<0.001). The interaction between sweeping velocity and water diversion rate on the number of fish entrained was not significant (*F*_2,35_ = 1.1, *P* = 0.340). There were no significant differences in body mass or fork length (*F*_6,35_ = 1.97, *P* = 0.096; *F*_6,35_ = 2.21, *P* = 0.065, respectively) among flow-combination groups and neither fork length nor body mass distinguished entrained and non-entrained fish (*P*≥0.340, t-tests), suggesting that within the range of juveniles tested all sizes were equally vulnerable to entrainment.

**Figure 3:**
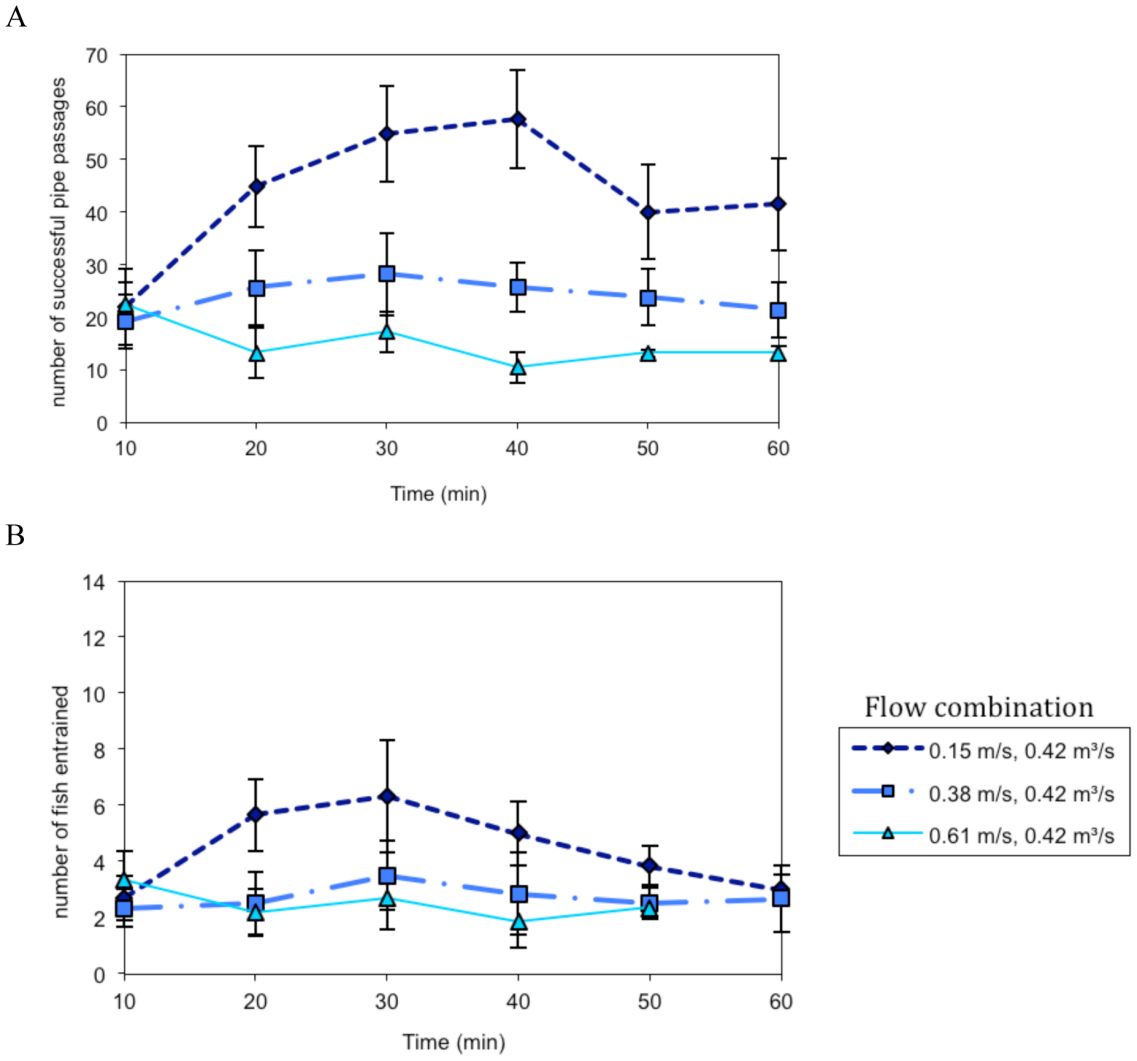
Mean ± SE number of fish that successfully passed the diversion pipe (A) or became entrained into the pipe (B) calculated in 10-min intervals at 0.15, 0.38 and 0.61 m/s sweeping velocities and a 0.42 m^3^/s water diversion rate.

**Figure 4:**
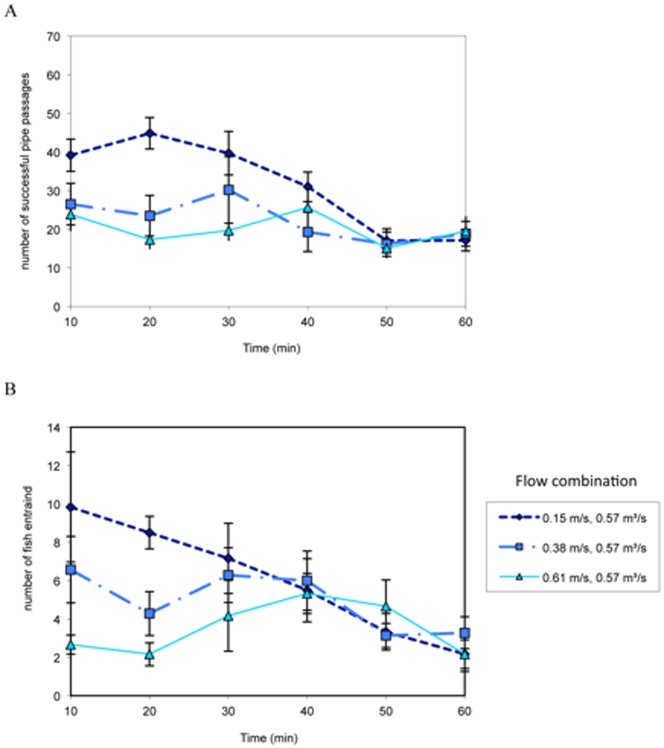
Mean ± SE number of fish that successfully passed the diversion pipe (A) or became entrained into the pipe (B) calculated in 10-min intervals at 0.15, 0.38 and 0.61 m/s sweeping velocities and a 0.57 m^3^/s water diversion rate.

In the 0.15 m/s sweeping velocity and 0.42 m^3^/s water diversion rate experiments the number of fish successfully traveling past the pipe (Figure 3A), and entrained into the pipe (Figure 3B), increased over time until the middle of the experiment, when fish passage began to decline. Pipe passage and entrainment rates were lower at the higher sweeping velocities, and remained fairly constant throughout the experimental period (Figure 3). In the 0.57 m^3^/s water diversion rate experiments (Figure 4), fish tested at the slowest sweeping flow had high passage and entrainment rates at the start of the experiment that declined over time. Successful pipe passage and entrainment rates were fairly consistent over time for fish tested at higher sweeping flows (Figure 4). Fish were rapidly (<1 s) drawn into the pipe and the majority of fish did not display behavioral escape responses (see Movie S1). Some fish were able to avoid being pulled into the pipe by quickly swimming to escape entrainment; the percentage of fish that escaped entrainment ranged from 5.4% at 0.15 m/s and 0.28 m^3^/s to 11.7% at 0.61 m/s and 0.42 m^3^/s.

**Figure 5:**
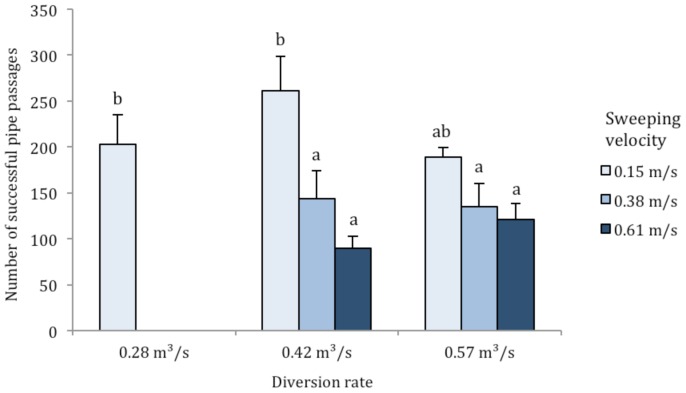
Mean ± SE number of green sturgeon that successfully passed the unscreened diversion pipe at sweeping velocities of 0.15, 0.38 and 0.61 m/s and water diversion rates of 0.28, 0.42 and 0.57 m^3^/s. Significant differences in the number of successful pipe passages at different flow combinations are marked with different letters (*P*≤0.039 for all significant pairwise comparisons).

**Figure 6:**
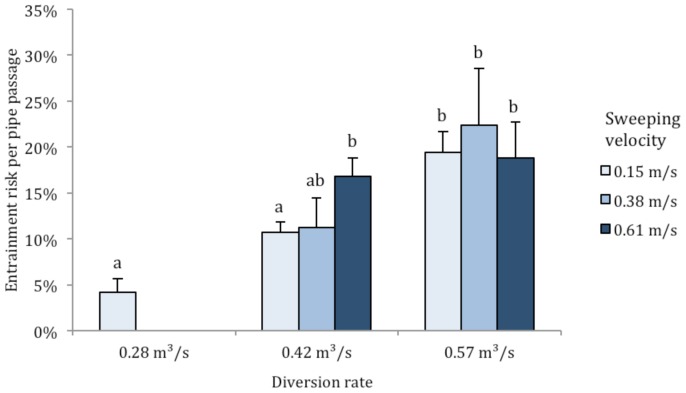
Calculated mean ± SE entrainment risk per pipe passage at sweeping velocities of 0.15, 0.38 and 0.61 m/s and water diversion rates of 0.28, 0.42 and 0.57 m^3^/s. Significant differences in the number of fish entrained among water diversion rates are marked with different letters (*P*≤0.006); fish entrainment did not significantly differ among sweeping velocities and there was no interaction.

**Figure 7:**
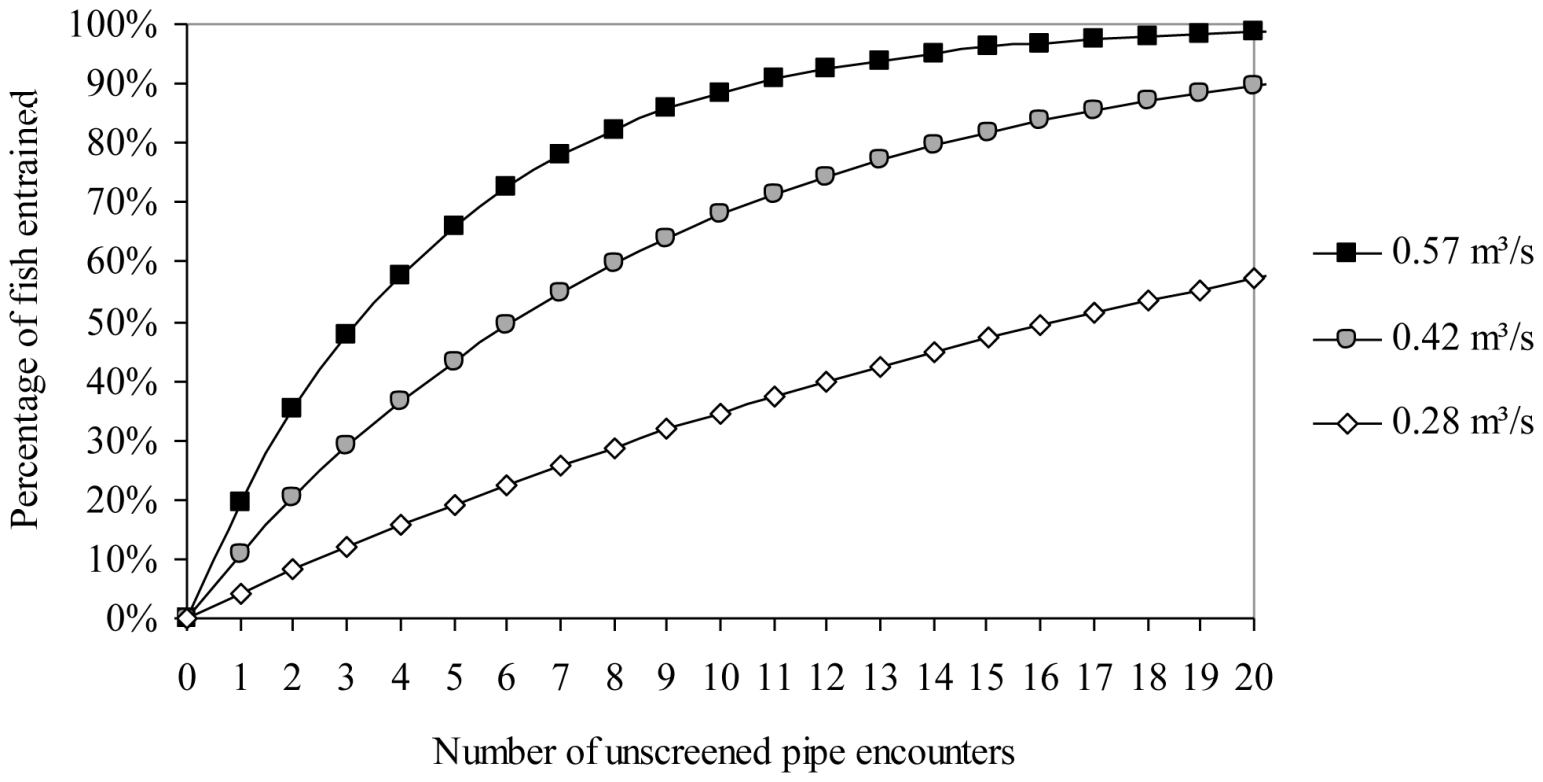
Laboratory-determined estimates of the percentage of juvenile green sturgeon lost to entrainment when repeatedly encountering (passing within 1.5 m of) unscreened pipes diverting 0.28 (▪), 0.42 (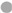), or 0.57 (◊) m^3^/s of water at a 0.15 m/s river (sweeping) velocity, calculated from entrainment risk per pipe passage values (Fig. 4).

Associated with their wider exploration of the flume, more fish successfully passed the diversion pipe at lower sweeping velocities (*F*_2,35_ = 11.0, *P*<0.001; Figure 5), pipe-passage rates were unaffected by water-diversion rates (*F*_2,35_ = 0.9, *P* = 0.401), and there was no interaction between sweeping velocity and diversion rate (*F*_2,35_ = 2.7, *P* = 0.084). Because more fish traveled past the water diversion pipe at slower sweeping velocities, we calculated entrainment risk on a per-passage basis to remove the influence of pipe-passage rates on entrainment. Overall, entrainment risk per pipe-passage ranged from 4.2% to 22.3% at different flow combinations (Figure 6). Although no significant differences were found among sweeping velocities (*F*_2,35_ = 0.56, *P* = 0.574), fish entrainment per pipe-passage significantly increased at higher water diversion rates (*F*_2,35_ = 12.46, *P*<0.001), and there was no interaction between sweeping velocity and diversion rate (*F*_2,35_ = 1.80, *P* = 0.180). The average percentage of fish entrained per pipe passage event increased with increasing water diversion rates at the 0.15 m/s sweeping velocity, from 4.2% at 0.28 m^3^/s, 10.6% at 0.42 m^3^/s, and 19.5% at 0.57 m^3^/s. Therefore fish entrainment increased by 364% when the water intake rate doubled (0.28 to 0.57 m^3^/s). Repeated encounters (swimming within 1.5 m of an active diversion pipe) with unscreened pipes seem likely, and we estimated that ≥50% of out-migrating fish could become entrained after encountering 3 to 16 unscreened pipes (at 0.57 to 0.28 m^3^/s diversion rates, respectively, Figure 7). The lack of behavioral avoidance may be related to the similar fish entrainment starting distance of 36.1 cm (SE, 0.7) from the center of the diversion pipe inlet, which was independent of sweeping velocities (*F*_2,32_ = 1.6, *P* = 0.123), water diversion rates (*F*_2,32_ = 2.3, *P* = 0.123), and their interaction (*F*_2,32_ = 3.3, *P* = 0.051). Thus, similar numbers of green sturgeon were entrained from upstream (44.8%) and downstream (55.2%) of the water-diversion pipe, although most fish were entrained from directly below (95.7%) the center of the pipe inlet (see Figures S1–S7 in File S1 for entrainment locations). Our flow probe was unable to measure water velocity in this central location, but at all other fish-entrainment locations mean water velocity was 0.50 m/s (SE, 0.04) and was independent of sweeping velocities (*F*_2,27_ = 2.7, *P*<0.089), water diversion rates (*F*_2,27_ = 1.8, *P*<0.179), and their interaction (*F*_2,27_ = 0.1, *P*<0.908).

## Discussion

Using a large-scale river simulation flume, we showed that juvenile green sturgeon are highly vulnerable to entrainment and loss through unscreened diversion pipes. These data indicate that in-river water diversions may represent a hazard to wild fish that, to date, has not been quantified and should be studied further. Results from previous studies testing how fish respond to variable hydraulic conditions are mixed with some species showing avoidance of accelerating water velocities while others show an attraction to accelerating flows [23], [24], [25], [26], [27]. Green sturgeon may have been able to move about the flume more easily at lower sweeping velocities, increasing pipe passage rates. Fish entrainment likely decreased at higher sweeping velocities because the fish would spend more time holding station in the current and less time exploring the flume. As the experiments progressed, fish likely acclimated to the flow and often began passing the pipe more frequently, resulting in increased entrainment and fewer fish in the flume, reducing pipe passage and entrainment rates in the second half of the experiments. Some fish species have been shown to avoid entering darkened structures [28], [29], but juvenile green sturgeon in our flume did not show a strong avoidance behavior when entering the modified hydraulic zone and darkened pipe inlet, increasing their odds for becoming entrained. Although we do not have a measure for how similar the fish act to passively drifting particles, which are influenced by discharge rates (Table 1), we know that they show a preference for the bottom and swim into the flow to hold their position, especially at the higher sweeping velocities. The variation in the behavioral response of fishes to flow field accelerations and hydraulic gradients has important implications for the development of any water diversion structure designed to guide fish safely past hazards or limit entrainment. Thus determining the response of juvenile green sturgeon to accelerating water flows is an important consideration for future study.

**Table 1:** Flume discharge rates at experimental sweeping velocities and diversion rates.

Sweeping Vel. (m/s)	Diversion rate (m^3^/s)	Discharge in the Flume (m^3^/s)
0.15	0.28	0.74
0.15	0.42	0.79
0.38	0.42	1.96
0.61	0.42	3.14
0.15	0.57	0.89
0.38	0.57	2.20
0.61	0.57	3.44

A second important consideration for future study is the development of laboratory methods to better quantify passage and entrainment *rates* as fish move in and out of zones of influence. Our analysis of the timing of successful passage and entrainment is imperfect because we could not track individual fish through time, repeated pipe encounters by the same individual were probable and variable, some fish were removed from the flume by entrainment through time, and differences in the duration of experimental exposure for fish remaining in the flume may have impacted their behavioral responses. Studies designed to quantify fish passage by tracking individual fish via telemetry have shown that rates of entrainment can vary, suggesting different passage mechanisms and performance despite the fact that an overall convergence in total entrainment values over time is possible [24]. In some instances, relying solely on total entrainment values and assuming that passage and entrainment rates are consistent through time can be misleading [30].

We have previously shown that juvenile Chinook salmon, *Oncorhynchus tshawytscha*, (ca. 13 cm fork length) tested in our experimental flume were susceptible to entrainment (with entrainment risk ranging from 0.3% to 2.3% when encountering the pipe, [16]), but to a much lesser degree in comparison to green sturgeon, where entrainment risk ranged from 4.2% to 22.3%. The low entrainment-avoidance rate in green sturgeon likely reflects a comparatively poor ability to detect the flow acceleration and directional changes near the pipe inlet at velocities ≥0.5 m/s (the average measured water velocity at entrainment starting positions). To our knowledge, burst swimming speeds have not been quantified in green sturgeon, but Allen et al. [14] has measured critical/endurance swimming velocities in juvenile green sturgeon in the size range tested here (ca. 350 mm FL versus 355 mm TL), and reported critical swimming velocities of 0.40–0.58 m/s, depending on ontogeny (sea-water tolerance). Therefore the velocities experienced by juvenile green sturgeon at the start of entrainment fall within their critical swimming speeds. Once an entrainment event started, 64% of the observed fish were rapidly drawn into the inlet, while the remainder increased swimming effort and escaped (see the online supplemental video for a visual demonstration of an entrainment event). A potential physiological explanation for this reduced perceptual capability, compared with juvenile Chinook salmon, is that sturgeon have few superficial neuromasts [31], the sensory organs that detect changes in water velocity and direction surrounding fish's bodies, which are frequently more numerous in other taxa [32]. Green sturgeon and sturgeon in general may have a lessened acute ability to detect water-diversion inflows compared to other fishes.

Water intake velocities generally increased with closer proximity to the pipe inlet and most sturgeon became entrained directly below the pipe inlet. This finding differed from previous findings for Chinook salmon where fish entrainments occurred at many depths, at both sides, and in front of the pipe inlet [16]. It is likely that the increased pipe-passage rates observed at low sweeping velocities, including passage through the modified hydraulic zone near the pipe's opening, increased entrainment rates in sturgeon. Coupled with their poor avoidance responses, increases in the number of times fish move past the water diversion pipe increases their entrainment risk. In contrast, at faster sweeping velocities, sturgeon held their locations in the flume by continuously swimming into the current rather than changing swimming direction and increasing passage rates. There was an exception to this pattern found in the 0.15 m/s sweeping velocity and 0.28 m^3^/s diversion rate combination, where the fish passage rate was generally high while few fish were entrained.

Green sturgeon ranging from 28–38 cm in fork length are known to be entrained into large agricultural water export facilities (State Water Project and Federal Central Valley Project) located in the San Francisco Bay Delta [5], indicating the presence of these fish in this system, but reliable enumeration that considers the impact of all potential green sturgeon mortality sources is lacking. The green sturgeon tested here (ca. 35 cm fork length) were within the size range entrained at the large government water projects, and suggest that these fish are susceptible to entrainment at smaller-scale unscreened water-diversion pipes. Norbriga *et al.* [33] sampled fish entrained through a 61-cm-diameter unscreened water diversion pipe at Horseshoe Bend in the lower Sacramento River with water intake rates of 0.4 to 1.0 m^3^/s, which produced similar intake velocities to those used in our study. During >66 h of sampling, unlike the simulation here, few fish greater than 3.5 cm were entrained through the diversion, although numerous inland silverside *Menidia beryllina* (3.0–5.0 cm) and striped bass *Morone saxatilis* (4.5–7.5 cm) were collected near the diversion using beach seines. Why these two smaller species avoided entrainment at similar intake velocities is uncertain. Possibly, striped bass and silverside may be more effective at detecting accelerating water velocities or changes in flow direction as compared to green sturgeon, and behaviorally avoided entrainment before the diversion velocity overwhelmed their swimming capabilities. The absence of green sturgeon in this field study [33] is unsurprising given the short sampling period and the rarity of green sturgeon in the Sacramento River, with estimates of only 10–28 individuals breeding annually [34]. Abundance estimates for immature sturgeon in the system are unknown.

While extrapolating entrainment risk from a laboratory study to a field situation is challenging, our data suggest that 22.3% of out-migrating juvenile green sturgeon could become entrained if they passed within 1.5 m of a single unscreened diversion pipe diverting 0.28 m^3^/s of water at a 0.15 m/s river (sweeping) speed. Moreover, if this rate were consistently observed at each diversion, 53% of out-migrating sturgeon could be lost to entrainment after encountering three unscreened water diversion pipes (Figure 7). These data suggest that the loss of migrating juvenile sturgeon to entrainment could be a significant but undetected source of green sturgeon mortality. However, we also show that green sturgeon entrainment risk is substantially decreased at a low water-diversion rate, and decreased intake velocity. Indeed, decreasing the water-diversion rate from 0.57 m^3^/s (19.5%) to 0.28 m^3^/s (4.2%) resulted in a 78% decrease in the number of fish entrained per pipe passage. For example, operating a 0.28 m^3^/s water diversion for twice as long, in order to divert an equivalent volume of water as a 0.57 m^3^/s diversion, would reduce the total number of entrained fish by more than half (57% reduction, calculated by ((4.2 *2)/19.5)-1). All the same, even at the lower 0.28 m^3^/s water diversion rate, ca. 50% of out-migrating green sturgeon would become entrained if they passed within 1.5 m of unscreened water diversions 16 times, suggesting that additional form(s) of behavioral or physical fish entrainment protection may be necessary to ensure safe passage, and thus reduce one possible source of mortality for these imperiled fish.

The entire known juvenile population of threatened Southern DPS green sturgeon exists only in the Sacramento River and Delta System. Our findings suggest that entrainment by the large number of unscreened agricultural water-diversion pipes located in this system poses a serious threat to the safe passage of these sturgeon, magnifying the ongoing threats to sturgeons in general. Green sturgeon juveniles may remain in fresh water for up to 1.5 years before entering seawater [15]. This residence time within the system likely results in juveniles experiencing numerous chance interactions with active water-diversion pipes, many of which we predict will be lethal. Placing agricultural diversion pipes near river bottoms, a common configuration designed to limit surfacing during dry or tidal periods as well as reduce contact with floating debris and vessels, potentially exacerbates entrainment risk by encroaching on the preferred benthic habitat of green sturgeon.

Decreasing water diversion rates could help juvenile green sturgeon safely pass by water diversion pipes, but any benefit depends on the local hydraulic conditions. Low water diversion rates and modifications that physically exclude fish from entering pipes (e.g. screens) or relocating pipe inlets to positions higher in the water column, may improve out-migration success. Our high estimates of juvenile green sturgeon entrainment susceptibility in a laboratory setting (relative to those estimated for Chinook salmon, for example see [16]) suggest that unscreened diversions could be a contributing mortality source for threatened Southern DPS green sturgeon. Future studies to determine the distribution of juvenile green sturgeon throughout the Sacramento River and Delta systems and listing the size, locations, and intake velocities of unscreened water diversion pipes are essential to accurately estimate the overall entrainment risk in a river setting. Studies of entrainment performance of green sturgeon of other sizes classes, under alternate experimental conditions (turbidity, day/night, temperature), and also using progeny from several families to evaluate any variation in sensory/behavior/swimming abilities associated with artificial culture should be considered to more fully determine the range of green sturgeon entrainment susceptibilities. Studies tracking the movements of individual fish could measure successful pipe passage distances and determine how fish respond during repeated pipe encounters. Our results should be interpreted with caution in linking laboratory results to in-river entrainment risk, but do suggest that new management strategies should be considered to balance agricultural needs in the Sacramento Valley with the conservation needs of these fish.

## Supporting Information

File S1 **Figures S1–S7.**
(DOC)

Movie S1 **Example video of juvenile green sturgeon entrained into the water diversion pipe during experiments at 0.15 m/s river (sweeping) velocity and 0.57 m^3^/s water diversion rate.**
(MOV)

## References

[1] Beamesderfer RCP, Farr RA (1997) Alternatives for the protection and restoration of sturgeons and their habitat. Environ Biol Fish 48: 407–417.

[2] Gessner J, Van Enennaam JP, Doroshov SI (1997) North American green and European Atlantic sturgeon: comparisons of life histories and human impacts. Environ Biol Fish 79: 397–4117.

[3] Birstein VJ, Bemis WE, Waldman JR (1997) The threatened status of acipenseriform species: a summary. Environ Biol Fish 48: 427–435.

[4] IUCN (2012) IUCN Red List of Threatened Species. Version 2012.1 Available: www.iucnredlist.org.

[5] Adams PB, Grimes C, Hightower JE, Lindley ST, Moser ML, et al (2007) Population status of North American green sturgeon, *Acipenser medirostris* . Environ Biol Fish 79: 339–356.

[6] Turnpenny AWH, Struthers G, Hanson KP (1998) A UK guide to intake fish-screening regulation, policy and best practice. Energy Technology Support Unit, Harwell. (www.thaaquatic.co.uk/publications.htm).

[7] Moyle PB, Israel JA (2005) Untested assumptions: effectiveness of screening diversions for conservation of fish populations. Fisheries 30: 20–29.

[8] Herren JR, Kawasaki SS (1997) Inventory of water diversions in four geographic areas in California's Central Valley. In Contributions to biology of Central Valley salmonids, Vol. 2. (ed. R. L. Brown), pp. 343–355. California Department of Fish and Game, Fish Bulletin 179, Sacramento, California. Available: http://www.dfg.ca.gov/fish/Resources/Reports/Bulletins.asp.

[9] Meier D (2013) US Fish and Wildlife Service, Director of the Anadromous Fish Screen Program, personal communication.

[10] Bowen M (2004) Inventory of barriers to fish passage in California coastal watersheds. The Coastal Conservancy Available: http://www.calfish.org/FishDataandMaps/IndependentDatasets/tabid/115/Default.aspx.

[11] Heublein JC, Kelly JT, Crocker CE, Klimley AP, Lindley ST (2009) Migration of green sturgeon, *Acipenser medirostris*, in the Sacramento River. Environ Biol Fish 84: 245–258.

[12] Kynard B, Parker E, Parker T (2009) Behavior of early life intervals of Klamath River green sturgeon, *Acipenser medirostris*, with a note on body color. Environ Biol Fish 72: 85–97.

[13] Vogel D (2011) Insights into the Problems, Progress, and Potential Solutions for Sacramento River Basin Native Anadromous Fish Restoration. (Prepared for Northern California Water Rights Associations and Sacramento Valley Water Users). Available: http://www.norcalwater.org/efficient-water-management/fisheries-enhancements/.

[14] Allen PJ, Cech JJ Jr (2007) Age/size effects on juvenile green sturgeon, *Acipenser medirostris*, oxygen consumption, growth, and osmoregulation in saline environments. Environ Biol Fish 79: 211–229.

[15] Allen PJ, Hobbs JA, Cech JJ Jr, Van Eenennaam JP, Doroshov SI (2009) Using trace elements in pectoral fin rays to assess life history movements in sturgeon: estimating age at initial seawater entry in Klamath River green sturgeon. Trans Am Fish Soc 138: 240–250.

[16] Mussen TD, Cocherell DE, Hockett Z, Ercan A, Bandeh H, et al (2013) Assessing juvenile Chinook salmon behavior and entrainment risk near unscreened water diversions: large flume simulations. Trans Am Fish Soc 142: 130–142.

[17] Van Eenennaam JP, Webb MAH, Deng X, Doroshov SI, Mayfield RB, et al (2001) Artificial spawning and larval rearing of Klamath River green sturgeon. Trans Am Fish Soc 130: 159–165.

[18] Deng X, Van Eenannaam JP, Doroshov SI (2002) Comparison of early life stages and growth of green and white sturgeon. Am Fish Soc 28: 237–248.

[19] Van Eenennaam JP, Linares-Casenave J, Doroshov SI (2012) Tank spawning of first generation domestic green sturgeon. J App Ichth 28: 505–511.

[20] Blumstein DT, Daniel JC, Evans CS (2006) *JWatcher 1.0 an introductory user's guide.* Available: http://www.jwatcher.ucla.edu/.

[21] Rasband WS (1997) ImageJ (U. S. National Institutes of Health, Bethesda, Maryland. Available: http://imagej.nih.gov/ij/).

[22] Bainbridge R (1958) The speed of swimming of fish as related to size and to the frequency and amplitude of the tail beat. J Exp Biol 35: 109–133.

[23] Enders EC, Gessel MH, Williams JG (2009) Development of successful fish passage structures for downstream migrants requires knowledge of their behavioural response to accelerating flow. Can J Fish Aquat Sci 66: 2109–2117.

[24] Haro A, Odeh M, Noreika J, Castro-Santos T (1998) Effect of water acceleration on downstream migratory behavior and passage of Atlantic salmon smolts and juvenile American shad at surface bypasses. Trans Am Fish Soc 127: 118–127.

[25] Kemp PS, Gessel MH, Sandford BP, Williams JG (2006) The behaviour of Pacific salmonid smolts during passage over two experimental weirs under light and dark conditions. River Res Applic 22: 429–440.

[26] Kemp PS, Gessel MH, Williams JG (2005) Fine-scale behavioral responses of Pacific salmonid smolts as they encounter divergence and acceleration of flow. Trans Am Fish Soc 134: 390–398.

[27] Vowles AS, Kemp PS (2012) Effects of light on the behaviour of brown trout (Salmo trutta) encountering accelerating flow: Application to downstream fish passage. Ecol Engineering 47: 247–253.

[28] Glass CW, Wardle CS (1995) Studies on the use of visual stimuli to control fish escape from codends. II. the effect of a black tunnel on the reaction behaviour of fish in otter trawl codends. Fish Res 23: 165–174.

[29] Kemp PS, Gessel MH, Williams JG (2005) Seaward migrating subyearling chinook salmon avoid overhead cover. J Fish Biol 67: 1381–1391.

[30] Castro-Santos T, Perry R (2012) Time-to-Event Analysis as a Framework for Quantifying Fish Passage Performance. Pages 427–452 in NS Adams, JW Beeman, JH Eilaer, editors. Telemetry techniques: a user guide for fisheries research. America Fisheries Society, Bethesda, Maryland.

[31] Gibbs MA, Northcutt RG (2004) Development of the lateral line system in the shovelnose sturgeon. Brain Behav Evol 64: 70–84. 15205543 10.1159/000079117

[32] Engelmann J, Hanke W, Mogdans J, Blechmann H (2000) Hydrodynamic stimuli and the fish lateral line. Nature 480: 51–52. 10.1038/35040706 11081502

[33] Nobriga ML, Matica Z, Hymanson ZP (2004) in *Early life history of fishes in the San Francisco estuary and watershed*, FFeyrer, L. RBrown, R. LBrown, J. JOrsi, Eds. (American Fisheries Society) pp. 281–295.

[34] Israel JA, May B (2010) Indirect genetic estimates of breeding population size in the polyploid green sturgeon (*Acipenser medirostris*). Mol Ecol 19: 1058–1070. 20149090 10.1111/j.1365-294X.2010.04533.x

