# Supplementary material for: Unscreened Water-Diversion Pipes Pose an Entrainment Risk to the Threatened Green Sturgeon, Acipenser medirostris
Source: PLoS One. 2014 Jan 15;9(1):e86321. doi: 10.1371/journal.pone.0086321 (PMC3893286; doi:10.1371/journal.pone.0086321)
Supplement: File S1 — Figures S1–S7. (DOC) [file pone.0086321.s001.doc]

Supplementary Materials:

**Supplemental Figures S1-S7**

A. Top view

0.15 m/s sweeping velocity and 0.28 m^3^/s diversion rate

B. Side view

C. Front view

● Fish entrainment starting location

○ Fish entrainment ending location

(pipe entrance position)

← Sweeping velocity direction

(the current is perpendicular to the

graphed plane in B. Side view)

▼ Top of the pipe inlet

**Figure S1.** Starting locations of green sturgeon entrainment events in the 0.15 m/s sweeping velocity and 0.28 m^3^/s diversion rate flow combination are plotted at three views of the diversion pipe: above the pipe (A), upstream view towards the pipe (B), into the pipe inlet (C). Locations where fish entered the pipe are also shown in (C). The 0-cm intercept for length (X), width (Y) and depth (Z) is located at the center of the pipe inlet in all three plots.

A. Top view

0.15 m/s sweeping velocity and 0.42 m^3^/s diversion rate

B. Side view

C. Front view

● Fish entrainment starting location

○ Fish entrainment ending location

(pipe entrance position)

← Sweeping velocity direction

(the current is perpendicular to the

graphed plane in B. Side view)

▼ Top of the pipe inlet

**Figure S2.** Starting locations of green sturgeon entrainment events in the 0.15 m/s sweeping velocity and 0.42m^3^/s diversion rate flow combination are plotted at three views of the diversion pipe: above the pipe (A), upstream view towards the pipe (B), into the pipe inlet (C). Locations where fish entered the pipe are also shown in (C). The 0-cm intercept for length (X), width (Y) and depth (Z) is located at the center of the pipe inlet in all three plots.

A. Top view

0.38 m/s sweeping velocity and 0.42 m^3^/s diversion rate

B. Side view

C. Front view

● Fish entrainment starting location

○ Fish entrainment ending location

(pipe entrance position)

← Sweeping velocity direction

(the current is perpendicular to the

graphed plane in B. Side view)

▼ Top of the pipe inlet

**Figure S3.** Starting locations of green sturgeon entrainment event in the 0.38 m/s sweeping velocity and 0.42 m^3^/s diversion rate flow combination are plotted at three views of the diversion pipe: above the pipe (A), upstream view towards the pipe (B), into the pipe inlet (C). Locations where fish entered the pipe are also shown in (C). The 0-cm intercept for length (X), width (Y) and depth (Z) is located at the center of the pipe inlet in all three plots.

A. Top view

0.61 m/s sweeping velocity and 0.42 m^3^/s diversion rate

B. Side view

C. Front view

● Fish entrainment starting location

○ Fish entrainment ending location

(pipe entrance position)

← Sweeping velocity direction

(the current is perpendicular to the

graphed plane in B. Side view)

▼ Top of the pipe inlet

**Figure S4.** Starting locations of green sturgeon entrainment events in the 0.61 m/s sweeping velocity and 0.42 m^3^/s diversion rate flow combination are plotted at three views of the diversion pipe: above the pipe (A), upstream view towards the pipe (B), into the pipe inlet (C). Locations where fish entered the pipe are also shown in (C). The 0-cm intercept for length (X), width (Y) and depth (Z) is located at the center of the pipe inlet in all three plots.

A. Top view

0.15 m/s sweeping velocity and 0.57 m^3^/s diversion rate

B. Side view

C. Front view

● Fish entrainment starting location

○ Fish entrainment ending location

(pipe entrance position)

← Sweeping velocity direction

(the current is perpendicular to the

graphed plane in B. Side view)

▼ Top of the pipe inlet

**Figure S5.** Starting locations of green sturgeon entrainment events in the 0.15 m/s sweeping velocity and 0.57 m^3^/s diversion rate flow combination are plotted at three views of the diversion pipe: above the pipe (A), upstream view towards the pipe (B), into the pipe inlet (C). Locations where fish entered the pipe are also shown in (C). The 0-cm intercept for length (X), width (Y) and depth (Z) is located at the center of the pipe inlet in all three plots.

A. Top view

0.38 m/s sweeping velocity and 0.57 m^3^/s diversion rate

B. Side view

C. Front view

● Fish entrainment starting location

○ Fish entrainment ending location

(pipe entrance position)

← Sweeping velocity direction

(the current is perpendicular to the

graphed plane in B. Side view)

▼ Top of the pipe inlet

**Figure S6.** Starting locations of green sturgeon entrainment events in the 0.38 m/s sweeping velocity and 0.57 m^3^/s diversion rate flow combination are plotted at three views of the diversion pipe: above the pipe (A), upstream view towards the pipe (B), into the pipe inlet (C). Locations where fish entered the pipe are also shown in (C). The 0-cm intercept for length (X), width (Y) and depth (Z) is located at the center of the pipe inlet in all three plots

A. Top view

0.61 m/s sweeping velocity and 0.57 m^3^/s diversion rate

B. Side view

C. Front view

● Fish entrainment starting location

○ Fish entrainment ending location

(pipe entrance position)

← Sweeping velocity direction

(the current is perpendicular to the

graphed plane in B. Side view)

▼ Top of the pipe inlet

**Figure S7.** Starting locations of green sturgeon entrainment events in the 0.61 m/s sweeping velocity and 0.57 m^3^/s diversion rate flow combination are plotted at three views of the diversion pipe: above the pipe (A), upstream view towards the pipe (B), into the pipe inlet (C). Locations where fish entered the pipe are also shown in (C). The 0-cm intercept for length (X), width (Y) and depth (Z) is located at the center of the pipe inlet in all three plots.
